# ROP16-Mediated Activation of STAT6 Suppresses Host Cell Reactive Oxygen Species Production, Facilitating Type III Toxoplasma gondii Growth and Survival

**DOI:** 10.1128/mBio.03305-20

**Published:** 2021-03-02

**Authors:** Joshua A. Kochanowsky, Kaitlin K. Thomas, Anita A. Koshy

**Affiliations:** a Department of Immunobiology, University of Arizona, Tucson, Arizona, USA; b BIO5 Institute, University of Arizona, Tucson, Arizona, USA; c Department of Neurology, University of Arizona, Tucson, Arizona, USA

**Keywords:** ROP16, STAT transcription factors, *Toxoplasma gondii*, innate immunity, reactive oxygen species

## Abstract

Polymorphic effector proteins determine the susceptibility of Toxoplasma gondii strains to IFN-γ-mediated clearance mechanisms deployed by murine host cells. However, less is known about the influence of these polymorphic effector proteins on IFN-γ-independent clearance mechanisms. Here, we show that deletion of one such polymorphic effector protein, ROP16, from a type III background leads to a defect in parasite growth and survival in unstimulated human fibroblasts and murine macrophages. Rescue of these defects requires a ROP16 with a functional kinase domain and the ability to activate a specific family of host cell transcription factors (STAT3, 5a, and 6). The growth and survival defects correlate with an accumulation of host cell reactive oxygen species (ROS) and are prevented by treatment with an ROS inhibitor. Exogenous activation of STAT3 and 6 suppresses host cell ROS production during infection with ROP16-deficient parasites and depletion of STAT6, but not STAT3 or 5a, causes an accumulation of ROS in cells infected with wild-type parasites. Pharmacological inhibition of NOX2 and mitochondrially derived ROS also rescues growth and survival of ROP16-deficient parasites. Collectively, these findings reveal an IFN-γ-independent mechanism of parasite restriction in human cells that is subverted by injection of ROP16 by type III parasites.

## Abstract (precis)

Toxoplasma gondii is an obligate intracellular parasite that infects up to one-third of the world’s population. Control of the parasite is largely accomplished by IFN-γ-dependent mechanisms that stimulate innate and adaptive immune responses.

## INTRODUCTION

For many microorganisms, differences in virulence between genetically similar strains are driven by polymorphisms that alter or enhance the activity of microbial proteins. Toxoplasma gondii, a common obligate intracellular pathogen capable of infecting most warm-blooded mammals, including humans, shows strain-specific differences in virulence (1–4). Acute and chronic infections are usually not harmful to immunocompetent individuals but can result in severe pathology and even death in the immunocompromised (4, 5). While limited evidence suggests that the genotype of the infecting strain can affect outcomes in humans, these strain-specific differences in outcomes are clearly established in mice (3, 5–13).

Historically, T. gondii strains were originally characterized into three clonal lineages (types I, II, and III) based on their acute virulence phenotype in mice (3, 14). Type I strains are highly virulent in laboratory mice, while type II strains exhibit an intermediate virulence, and type III strains are considered avirulent (14). Of the canonical strains, types I and III are more genetically similar compared to type II. Advances in sequencing capabilities have led to T. gondii strains now falling into 15 genetic haplotypes of which type I, type II, and type III are now called haplotypes 1, 2, and 3 and clades A, D, and C, respectively (14, 15). Our knowledge of how type I, II, and III strains cause distinct acute disease outcomes in mice has been mapped to differences in the proteins that the parasite secretes into its host cell during and after the invasion process (16–18). These studies also identified several polymorphic proteins that seemed to have no role in lethality of type I strains in mice (19–21). One possibility for the role of these polymorphic proteins is that these strain-specific differences are critical to parasite survival in different, yet-to-be-identified hosts (16). Another possibility is that some of these proteins do not influence disease outcomes of the hypervirulent type I strains but may be critical in the less virulent strains.

One such polymorphic effector protein is rhoptry protein 16 (ROP16). ROP16s from types I, II, and III translocate into the host cell nucleus and have a functional kinase domain, but only the type I and III alleles, which are 99% identical in their amino acid sequence, cause prolonged phosphorylation and activation of STAT3 and 6 and potentially STAT5a (21–24). A single amino acid change in the type II allele of ROP16 renders it incapable of STAT activation (24). In macrophages, the ability of the type I allele of ROP16 to cause prolonged activation of STAT3/6 leads to decreased production of IL-12, a key proinflammatory cytokine involved in generating the IFN-γ response (21, 22). In the context of highly virulent type I strains, ROP16 has only a minimal effect on pathogenesis in mice (21), but ROP16’s role in avirulent type III strains has been understudied.

Our group and another recently demonstrated that ROP16 plays a key role in determining the acute and central nervous system (CNS) immune response in mice and is required for efficient type III parasite persistence in mice, possibly by regulating the early immune response (25, 26). During that work, we also identified an IFN-γ-independent *in vitro* growth defect in human fibroblasts in a type III strain that lacked ROP16 (IIIΔ*rop16*). Given this IFN-γ-independent phenotype, we hypothesized that different mechanisms accounted for the *in vivo* and *in vitro* survival defects of the IIIΔ*rop16* strain. Here, we expand on those initial findings by complementing the IIIΔ*rop16* parasites with a series of ROP16 mutants that lack different ROP16 domains/functions. These experiments determined that efficient *in vitro* growth required a functional kinase domain and the ability to phosphorylate STATs. Consistent with these data, the activation of STAT3/6 via cytokine stimulation was sufficient to restore parasite growth in IIIΔ*rop16* parasites. Given the recent finding that type III strains are killed in naive murine macrophages in a reactive oxygen species (ROS)-dependent manner (27), we sought to determine if ROP16 was influencing host cell ROS production in human cells. Indeed, we found that host cells infected with IIIΔ*rop16* parasites accumulated ROS and that pharmacological inhibition of NADPH oxidase 2 (NOX2) and mitochondrially derived ROS restored IIIΔ*rop16* parasites growth to wild-type levels. Finally, using short hairpin RNA (shRNA) knockdown cells and mouse ear tip fibroblasts (mETFs) derived from STAT6 knockout (STAT6KO) mice, we determined that activation of STAT6 was needed for suppression of host cell ROS production and efficient parasite growth and survival. These findings highlight a mechanism by which type III T. gondii strains evade IFN-γ-independent cell-autonomous defenses in human and murine cells.

## RESULTS

### Generation and characterization of IIIΔ*rop16* and ROP16 domain mutants.

**FIG 1:**
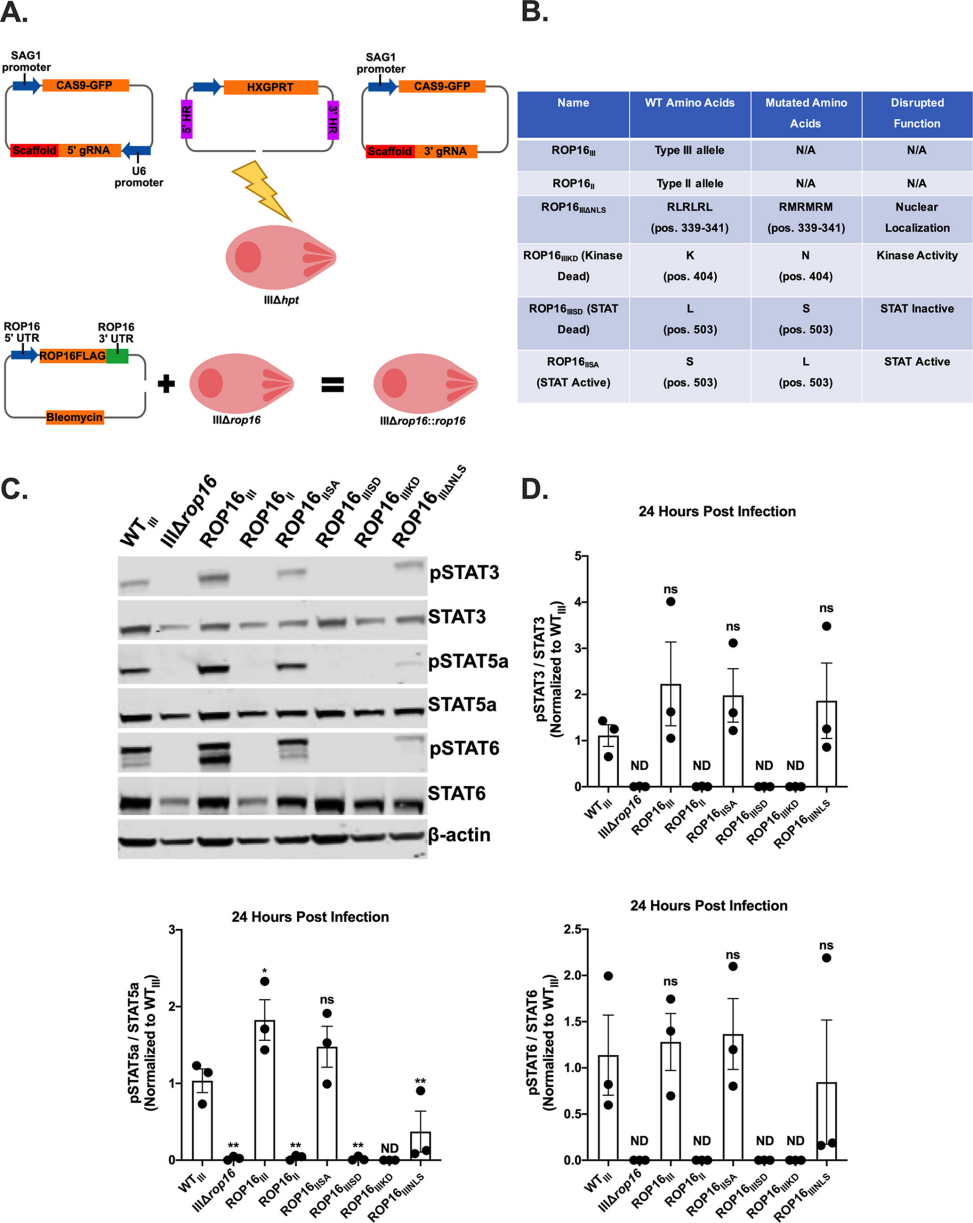
Characterization of ROP16 constructs. (A) Schematic depicting generation of ROP16-expressing strains. Type IIIΔ*hxgprt* parasites were transfected with CRISPR/CAS9 vectors targeting 500 bp upstream (gRNA Up) and downstream (gRNA Down) of the *rop16* coding sequence and a linearized vector with 500-bp regions of homology (HR) to the 5′ and 3′ untranslated regions (UTRs) of *rop16* surrounding a selectable marker, hypoxanthine-xanthine-guanine phosphoribosyl transferase (HXGPRT). Complementation was achieved using a linearized vector encoding a FLAG-tagged ROP16 construct and a selectable bleomycin resistance marker. (B) Table listing mutations and nomenclature for the ROP16-complemented strains. (C) Representative Western blots from HFFs infected with the identified strains (MOI 5). Protein isolation was done at 24 h postinfection (hpi). (D) Graphs of the densitometry of phosphorylated STAT3/5a/6 normalized to total STAT3/5a/6 Western blots. Bars, mean ± SEM. Each dot = 1 experiment, *n* = 3 experiments. One-way ANOVA, Dunnett’s multiple-comparison test compared to WT_III_. *, *P* ≤ 0.05; **, *P* ≤ 0.005. ND = not detected. ns = not significant.

We previously generated a ROP16-deficient type III strain (IIIΔ*rop16*) and complement (ROP16_III_) using a CRISPR/Cas9-based approach (Fig. 1A) (25). We noted that the IIIΔ*rop16* strain showed an *in vitro* IFN-γ-independent growth defect, while the *in vivo* deficit related to murine IFN-γ-dependent mechanisms, suggesting that ROP16 might enhance type III parasite fitness in multiple ways (25, 26). To better define this *in vitro* growth defect, we sought to identify which functional domains of ROP16 were required for efficient parasite growth. To accomplish this goal, we generated a series of IIIΔ*rop16* complemented strains, which expressed the type II ROP16 (ROP16_II_), type III ROP16 (ROP16_III_), or domain mutants that were catalytically inactive (ROP16_IIIKD_) or lacked a nuclear localization sequence (ROP16_IIIΔNLS_) (Fig. 1B). Finally, as previous work had demonstrated that a single polymorphic amino acid on ROP16 determined the strain-specific activation of STAT3 and was predicted to determine the activation of STAT5a and 6, we also generated complemented strains that expressed a ROP16_III_ in which the leucine at position 503 was switched to a serine, rendering it “STAT-dead” (ROP16_IIISD_), or expressed a ROP16_II_ in which the serine at position 503 was changed to a leucine, rendering it “STAT-active” (ROP16_IISA_) (Fig. 1B) (24). We validated the deletion and reintroduction of *rop16* in our transgenic strains using a locus-specific PCR that amplifies a 1,000-bp product in the coding sequence of *rop16* (see Fig. S1A in the supplemental material). We confirmed expression of our ROP16 constructs via immunofluorescent (IFA) staining of their engineered FLAG tag (Fig. S1A and B). Finally, we confirmed that our ROP16_IIIΔNLS_-expressing strain was unable to translocate to the nucleus of the host cell (Fig. S1C).

10.1128/mBio.03305-20.1 FIG S1 Confirmation of ROP16 expression. (A) PCR of *rop16* locus for identified strains. PCR analysis of SAG1 was used as a DNA control. (B) Immunofluorescence for FLAG-tagged ROP16 constructs. HFFs were infected with identified strains (MOI 1) for 24 h. Images depict ROP16-FLAG tag (green), SAG1 (red), and nuclei (DAPI stain, blue). (C) Immunofluorescence for nuclear localization of FLAG-tagged ROP16 constructs. HFFs were infected with ROP16_III_ and ROP16_IIIΔNLS_ (MOI 5) for 6 h. Images depict ROP16-FLAG tag (green), SAG1 (red), and nuclei (DAPI stain, blue). Arrows indicate FLAG signal in the nucleus of infected cells. Download FIG S1, EPS file, 1.1 MB. Copyright © 2021 Kochanowsky et al. 2021 Kochanowsky et al. https://creativecommons.org/licenses/by/4.0/ This content is distributed under the terms of the Creative Commons Attribution 4.0 International license.

As most work has been done with the type I ROP16 allele, we first sought to confirm that the type III allele also causes prolonged activation of STAT3, 5a, and 6. To that end, we analyzed human foreskin fibroblasts (HFFs) infected with our panel of engineered type III strains (Fig. 1B). As expected, given that the type III allele is 99% identical at the amino acid level (3 to 6 amino acid differences) to the type I allele, wild-type (WT_III_) and ROP16_III_ parasites induced prolonged phosphorylation of STAT3, 5a, and 6 while the IIIΔ*rop16*, ROP16_II_, and ROP16_IIIKD_ strains did not (Fig. 1C and D). Additionally, we found that the single polymorphic amino acid in ROP16 that determines the strain-specific activation of STAT3 also determines activation of STAT5a and STAT6 (Fig. 1C and D). Consistent with prior studies, the lack of an NLS had no effect on STAT3 and 6 phosphorylation but significantly decreased STAT5a phosphorylation (Fig. 1C and D).

Together, these data show that the type III allele of *rop16* is a functional kinase capable of activating STAT3, 5a, and 6. Additionally, the leucine at position 503 dictates the ability of ROP16 to activate not only STAT3 but also STAT5a and STAT6. Finally, mutation of the NLS in ROP16 leads to decreased phosphorylation of STAT5a, but not STAT3 or STAT6.

### ROP16 facilitates parasite growth and survival.

**FIG 2:**
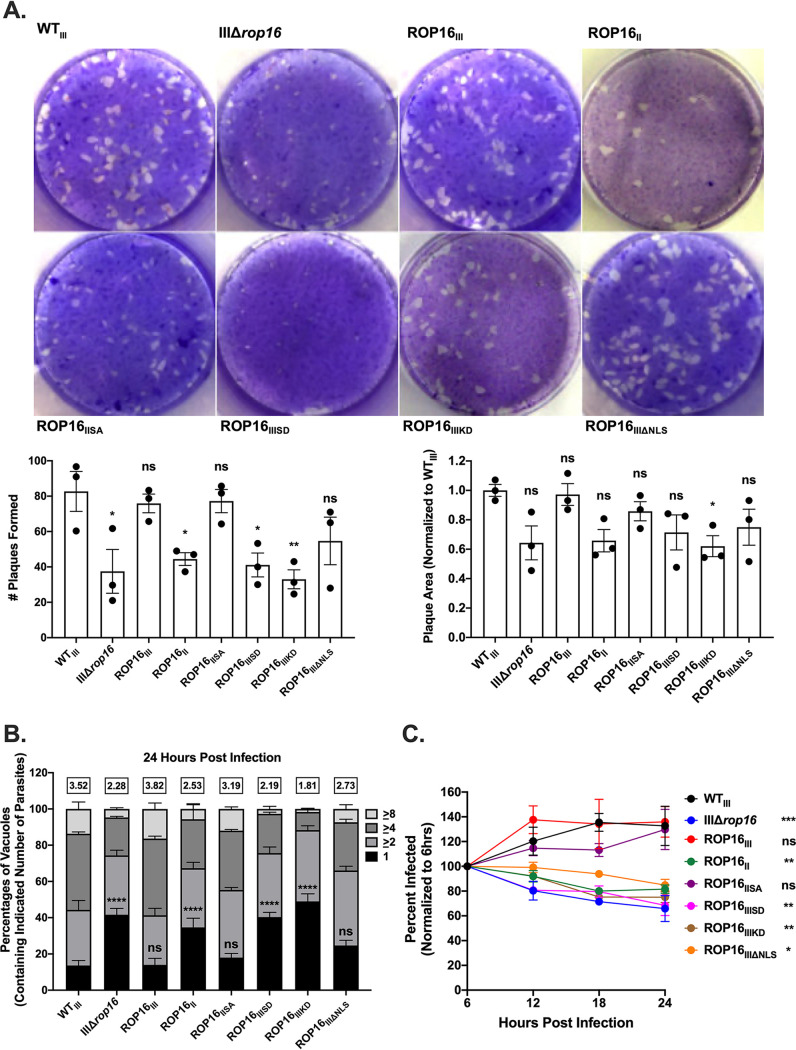
Efficient type III growth and survival requires a ROP16 with an active kinase domain and a leucine at position 503. (A) (Top) Representative images of plaque assays at 10 days postinfection (dpi). HFFs were initially infected with 200 parasites of the identified strains. (Bottom left) Quantification of plaques formed by identified strains. (Bottom right) Quantification of plaque area relative to WT_III_. Each dot = 1 experiment, *n* = 3 experiments. Bars, mean ± SEM. (B) Replication of identified strains at 24 hpi. Percentages of parasite vacuoles containing indicated number of parasites. Numbers above stacked bar graph represent the overall mean number of parasites per vacuole. Bars, mean ± SEM. *n* ≥ 100 vacuoles/well, 3 wells/experiment, 3 experiments. (C) Intracellular survival of identified strains in HFFs shown as percentage of initial infection at 6 hpi. HFFs were infected with parasites (MOI 1) and, at appropriate time points, fixed and stained for SAG1 to detect parasites and wheat germ agglutinin (WGA) to define host cells. Values, mean ± SEM. *n* = 3 experiments. (A and C) One-way ANOVA. Dunnett’s multiple-comparison test compared to WT_III_. *, *P* ≤ 0.05; **, *P* ≤ 0.005; ***, *P* ≤ 0.0005. ns = not significant. (B) Two-way ANOVA, Dunnett’s multiple-comparison test compared to WT_III_. ****, *P* ≤ 0.0001. ns = not significant.

We previously reported that IIIΔ*rop16* parasites were defective in plaque production *in vitro*, but not in invasion and attachment, indicating a possible defect in parasite growth and survival (25). To further characterize this defect, we performed a plaque assay using our newly generated panel of ROP16 parasites. IIIΔ*rop16*, ROP16_II_, ROP16_IIISD_, and ROP16_IIIKD_ parasites were all defective in forming plaques compared to WT_III_ parasites. ROP16_III_, ROP16_IISA_, and ROP16_IIIΔNLS_ parasites formed plaques at levels comparable to WT_III_ (Fig. 2A).

To test whether this defect in plaque formation was at the level of parasite growth, we infected confluent monolayers of HFFs with our parasite strains and then enumerated the number of parasites per parasitophorous vacuole (PV) at 24 h postinfection (hpi). We found that the ROP16_II_, ROP16_IIISD_, and ROP16_IIIKD_ strains displayed an accumulation of PVs that contained only one parasite compared to WT_III_ parasites, indicating an overall growth defect, while parasites expressing ROP16_III_, ROP16_IISA_, or ROP16_IIIΔNLS_ had WT_III_ levels of growth (Fig. 2B).

As a recent study demonstrated that type III strains are cleared from naive murine bone marrow-derived macrophages (BMDMs) without the need for IFN-γ stimulation (27), we decided to test if our ROP16 strains were being cleared in HFFs. To this end, we infected confluent monolayers of HFFs with our parasites and then fixed and counted the number of infected cells every 6 h for 24 h. We observed a steady accumulation of the percentage of infected cells over 24 h in our WT_III_, ROP16_III_, and ROP16_IISA_ strains but noticed a significant decrease in the number of infected host cells in our ROP16_II_, ROP16_IIISD_, and ROP16_IIIKD_ strains (∼15 to 20% on average) over time, indicating that parasites were being cleared in an IFN-γ-independent manner (Fig. 2C). The ROP16_IIIΔNLS_ strain showed a significant but less pronounced clearance in HFFs (Fig. 2C).

Together, these data indicate that ROP16 is needed for efficient parasite growth and survival *in vitro*. Efficient parasite growth and survival *in vitro* are dependent on kinase activity, and the ability of ROP16_IISA_ to restore these defects suggests that activation of STATs is necessary as well. Interestingly, while the ROP16_IIIΔNLS_-expressing strain had no growth defect, there was a small but significant increase in parasite clearance, indicating that nuclear localization of ROP16 may play a limited role in parasite survival. Finally, we expanded on recent work (25–27) by showing that ROP16 suppresses clearance of type III parasites in unstimulated human fibroblasts.

### Type III parasites accumulate ROS in their host cells in a ROP16-dependent manner.

IFN-γ exerts its antiparasitic effects at the cellular level by upregulating immunity-related GTPases (IRGs) and guanylate binding proteins (GBPs) that disrupt the PV and lead to eventual clearance of the parasite in mice (28–34). Several other studies have also implicated the IFN-γ-dependent production of nitric oxide and autophagy-related clearance (21, 35, 36). Given that human cells lack the same IRG systems and our observation of an IFN-γ-independent defect in parasite growth and survival, we reasoned that the mechanism of parasite suppression and clearance *in vitro* must be something other than these canonically described IFN-γ-dependent mechanisms.

Prior work demonstrated that host cells infected with type I parasites deficient in ROP16 showed an accumulation of reactive nitrogen species (RNS) in an IFN-γ-dependent manner (21). Given that the type III strain has shown significant differences from the type I strain (25, 26), we sought to determine if IFN-γ-independent RNS production might also be playing a role in the IIIΔ*rop16* survival defect by quantifying RNS levels in host cells. We observed no difference in RNS accumulation among any of our strains in our unstimulated HFFs, indicating that IFN-γ-independent RNS production is not playing a role in the ROP16-dependent clearance we see in HFFs (Fig. S2A).

**FIG 3:**
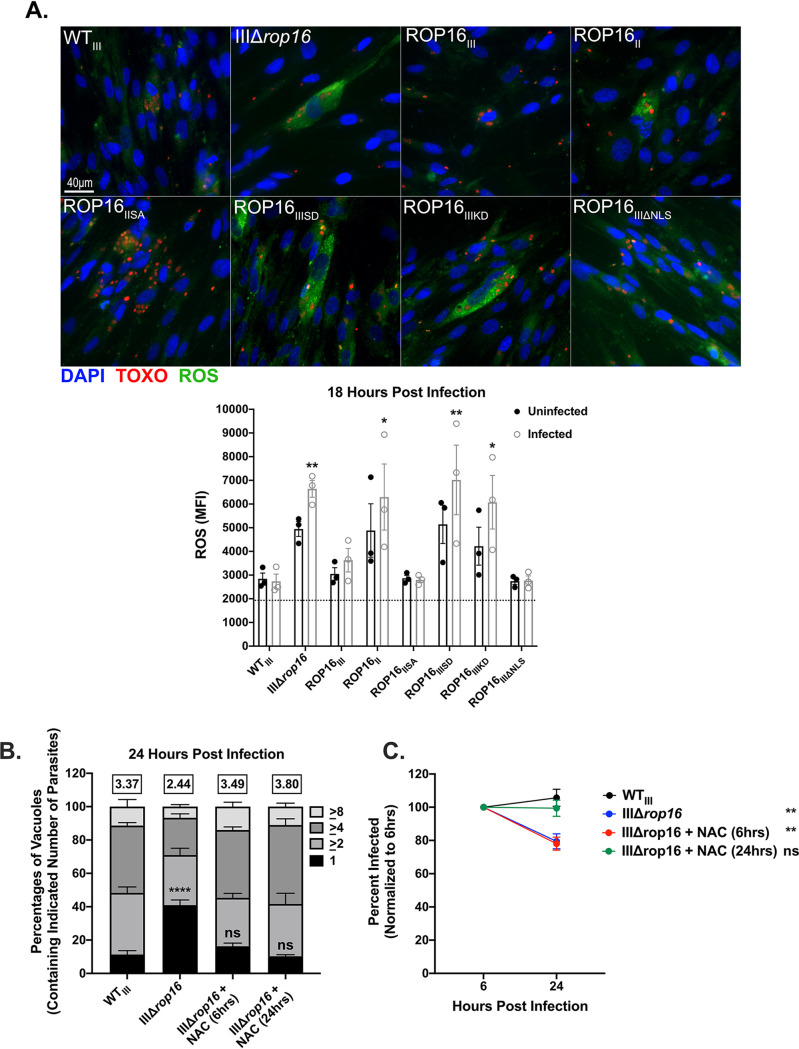
Host cell reactive oxygen species (ROS) restrict intracellular parasites in a ROP16-dependent manner. (A) Representative images of ROS accumulation at 18 hpi in HFFs infected with the identified strains (MOI 1). ROS (green), parasites (red), and nuclei (DAPI stain, blue). Bottom, quantification of ROS. Each dot = mean ROS value for 1 experiment in the indicated group. *n* = 100 cells/experiment, 3 experiments. Bars, mean ± SEM. Black dotted line indicates the mean ROS value of uninfected controls. MFI, mean fluorescence intensity. (B) Replication of indicated strain in HFFs ± treatment with a ROS inhibitor, N-acetyl-l-cysteine (NAC), for 6 or 24 h during infection. Mean percentages of parasite vacuoles containing indicated number of parasites. Numbers above stacked bar graph represent the overall mean number of parasites per vacuole. Bars, mean ± SEM. *n* ≥ 100 vacuoles/well, 3 wells/experiment, 3 experiments. (C) Intracellular survival of identified strain ± treatment with NAC for 6 or 24 h during infection in HFFs expressed as a percentage of initial infection at 6 hpi. HFFs were infected with parasites (MOI 1) and stained as described in the legend to Fig. 2. Values, mean ± SEM. *n* = 3 experiments. (A and C) One-way ANOVA, Dunnett’s multiple-comparison test compared to WT_III_. *, *P* ≤ 0.05, and **, *P* ≤ 0.005. ns = not significant. (B) Two-way ANOVA, Dunnett’s multiple-comparison test compared to WT_III_. ****, *P* ≤ 0.0001. ns = not significant.

10.1128/mBio.03305-20.2 FIG S2 Effect of ROP16 on reactive nitrogen species (RNS) and GBP5 accumulation in HFFs. (A) Quantification of host cell accumulation of RNS. HFFs were infected with indicated strains and analyzed as in Fig. 3 except now quantifying for RNS signal. Each dot = RNS value for 1 cell. *n* = 100 cells, 1 experiment. Bars, mean ± SD. Black dotted line indicates the mean RNS value of uninfected controls. (B) Quantification of GBP5 localization around the PV at 9 hpi. Cells were treated with or without 100 U of IFN-γ for 24 h prior to infection and then infected with the indicated strains for 9 h. Cells were stained as described in Materials and Methods, and localization of GBP5 to the PV was quantified. Each dot = mean number of parasites that stained positive for GBP5 per experiment. *n* = 100 PVs counted over 50 fields of view per experiment, 3 experiments. Bars, mean ± SEM. Download FIG S2, TIF file, 0.5 MB. Copyright © 2021 Kochanowsky et al. 2021 Kochanowsky et al. https://creativecommons.org/licenses/by/4.0/ This content is distributed under the terms of the Creative Commons Attribution 4.0 International license.

As a recent study demonstrated that type III parasites are cleared by ROS-dependent and GBP5-dependent mechanisms in naive murine BMDMs (27), we hypothesized that these mechanisms might be involved in the restriction and clearance of IIIΔ*rop16* parasites in HFFs. To test whether GBP5 was involved, we infected confluent monolayers of HFFs, treated with or without IFN-γ, with our parasite strains and quantified the accumulation of GBP5 around parasite-containing vacuoles. At 9 hpi, we detected little GBP5 accumulation around the PV (1 to 3%) regardless of ROP16 status in untreated HFFs (Fig. S2B). Prestimulation with IFN-γ resulted in an increase in GBP5 accumulation around PVs, but ROP16 status did not affect GBP5 localization to the PV (Fig. S2B).

Finally, to test whether ROS played a role in parasite restriction, we infected confluent monolayers of HFFs with our parasite strains, and at 18 hpi, we quantified host cell ROS accumulation via IFA. In cells infected with the IIIΔ*rop16*, ROP16_II_, ROP16_IIISD_, and ROP16_IIIKD_ strains, we observed an increased accumulation of ROS compared to cells infected with WT_III_ parasites (Fig. 3A). Though not statistically significant (*P* value = 0.3163, 0.3505, 0.2217, and 0.7847, respectively), the uninfected cells in these cultures showed a trend toward an increased accumulation of ROS compared to uninfected cells in cultures with WT_III_ parasites. In ROP16_III_-, ROP16_IISA_-, and ROP16_IIIΔNLS_-infected cultures, infected and uninfected host cells displayed similar levels of ROS accumulation as WT_III_-infected cultures (Fig. 3A).

Given the accumulation of host cell ROS, but not RNS or GBP5, in cultures infected with IIIΔ*rop16* parasites, we hypothesized that ROS was the major driver of the growth and survival defects in our IIIΔ*rop16* strain. To test this hypothesis, we pharmacologically inhibited ROS production using a ROS inhibitor, N-acetyl-l-cysteine (NAC), during infection and then quantified parasite growth and survival. Consistent with ROS driving the IIIΔ*rop16* defects, treatment of HFFs with NAC for 6 or 24 h was sufficient to restore growth of IIIΔ*rop16* parasites to WT_III_ levels (Fig. 3B). However, only 24-h treatment of NAC was sufficient to restore survival of IIIΔ*rop16* parasites (Fig. 3C). NAC treatment during IIIΔ*rop16* infection was also sufficient to reduce ROS to levels comparable to HFFs infected with WT_III_ (Fig. S3). Taken together, these data demonstrate that ROP16-dependent suppression of ROS accumulation is a key mechanism used by type III parasites to avoid clearance by host cells.

10.1128/mBio.03305-20.3 FIG S3 Effect of NAC treatment on ROS accumulation during parasite infection. Quantification of ROS as analyzed in Fig. 3 in HFFs ± treatment with NAC for 24 h during infection with indicated strains. Each dot = ROS value for 1 cell. *n* = 100 cells, 1 experiment. Bars, mean ± SD. Black dotted line indicates the mean ROS value of uninfected controls. One-way ANOVA. Dunnett’s multiple-comparison test compared to WT_III_. ****, *P* ≤ 0.0001. ns = not significant. Download FIG S3, TIF file, 0.3 MB. Copyright © 2021 Kochanowsky et al. 2021 Kochanowsky et al. https://creativecommons.org/licenses/by/4.0/ This content is distributed under the terms of the Creative Commons Attribution 4.0 International license.

### ROS-mediated clearance of IIIΔ*rop16* also occurs in murine macrophage-like cell lines.

**FIG 4:**
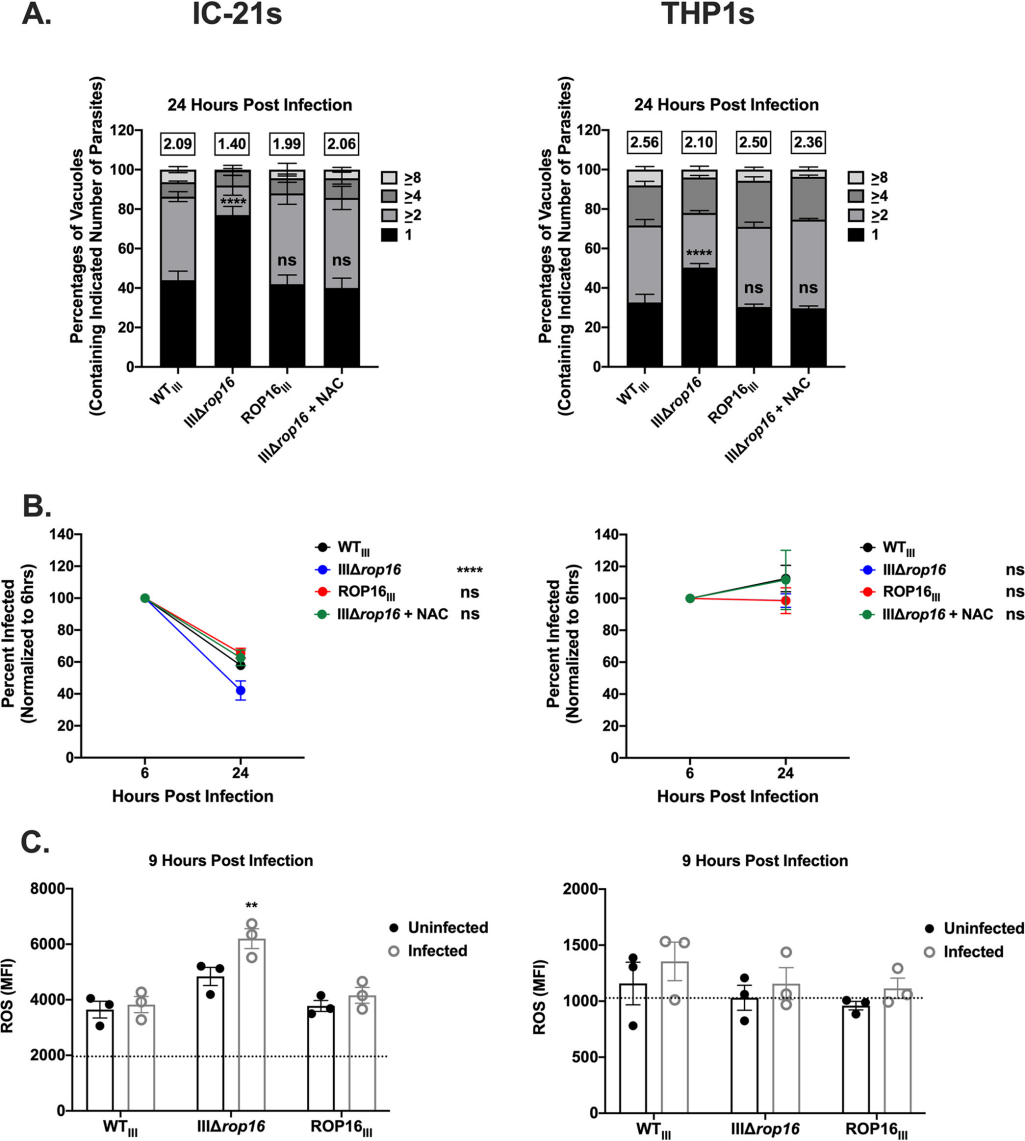
Parasite growth and survival is restricted by ROS in IC-21s but not in differentiated THP1s. (A) Replication of indicated strains in IC-21s and differentiated THP1s ± treatment with NAC for 24 h during infection. Mean percentages of parasite vacuoles containing indicated number of parasites. Numbers above stacked bar graph represent the overall mean number of parasites per vacuole. Bars, mean ± SEM. *n* ≥ 100 vacuoles/well, 3 wells/experiment, 3 experiments. (B) Intracellular survival of indicated strains *±* treatment with NAC for 24 h during infection of IC-21s and differentiated THP1s expressed as a percentage of initial infection at 6 hpi. HFFs were infected with parasites (MOI 1) and stained as described in the legend to Fig. 2. (C) Quantification of ROS as in Fig. 3. Each dot = mean ROS value for 1 experiment in the indicated group. *n* = 100 cells/experiment, 3 experiments. Bars, mean ± SEM. Black dotted line indicates the mean ROS value of uninfected controls. (A) Two-way ANOVA, Dunnett’s multiple-comparison test compared to WT_III_. ****, *P* ≤ 0.0001. ns = not significant. (B and C) One-way ANOVA, Dunnett’s multiple-comparison test compared to WT_III_. **, *P* ≤ 0.005, and ****, *P* ≤ 0.0001. ns = not significant.

We next sought to determine if ROP16-mediated ROS accumulation restricted parasites in mouse and human macrophage cell lines. We infected either IC-21s, a mouse macrophage-like cell line (37), or THP1s, a human monocyte cell line (38) that converts to macrophages when treated with 12-O-tetra-decanoylphorbol-13-acetate (TPA) (39), with WT_III_, IIIΔ*rop16*, or ROP16_III_ parasites and measured parasite growth and survival. Similar to our results in HFFs, IIIΔ*rop16* parasites exhibited a growth defect compared to WT_III_ parasites that could be restored with a 24-h treatment of NAC in both cell lines (Fig. 4A). While infection of IC-21s with WT_III_, IIIΔ*rop16*, or ROP16_III_ resulted in a decrease in parasite survival, only IIIΔ*rop16* exhibited an increased clearance in this cell type compared to WT_III_, and this increased clearance could be inhibited by treatment with NAC (Fig. 4B). Interestingly, despite IIIΔ*rop16* exhibiting a growth defect in differentiated THP1s, there was no effect on parasite survival for any of the indicated strains (Fig. 4B). Finally, we quantified ROS accumulation in these cell lines during infection. In keeping with previous results, IC-21s infected with IIIΔ*rop16* parasites had an increased amount of ROS compared to WT_III_, while differentiated THP1s infected with IIIΔ*rop16* did not (Fig. 4C). Together, these results indicate that ROP16-dependent suppression of ROS accumulation occurs in murine macrophage-like cells but not in human macrophages differentiated from a monocyte cell line, suggesting that ROP16 effects may vary between hosts and cell types.

### Suppression of STAT6 results in accumulation of ROS and a defect in type III parasite growth and survival.

Given the ability of ROP16_IISA_ but not ROP16_IIISD_ to restore parasite growth and survival, our data suggested, but did not directly show, that STAT activation was involved in suppressing host cell ROS production. To determine if STAT activation was needed for parasite growth and survival, we prestimulated HFFs with either IL-4 or IL-6 (40–44). Prestimulation of HFFs with either IL-4 or IL-6 resulted in the activation of STAT3 and 6, but not STAT5a (Fig. S4A). Additionally, IL-4 or IL-6 prestimulation of HFFs fully restored growth of IIIΔ*rop16* to WT_III_ levels but only partially restored parasite survival (Fig. S4B and C). Together, these data indicate that STAT3 and 6 may be required for type III parasite growth and survival, while STAT5a may play a less prominent role.

10.1128/mBio.03305-20.4 FIG S4 IL-4 and IL-6 stimulation restores parasite growth and survival. (A) Representative Western blot from HFFs infected with WT_III_ strain (MOI 5) or stimulated with IL-4 or IL-6 (100 ng). Protein isolation was done at 24 hpi for infected HFFs or at 6 h poststimulation for cytokine-stimulated HFFs. (B) Replication at 24 hpi of indicated strains in HFFs ± treatment with IL-4 or IL-6 (100 ng). Mean percentages of parasite vacuoles containing indicated number of parasites. Numbers above stacked bar graph represent the overall mean number of parasites per vacuole. Bars, mean ± SEM. *n* ≥ 100 vacuoles/well, 3 wells/experiment, 3 experiments. (C) Intracellular survival at 24 hpi of indicated strains in HFFs ± treatment with IL-4 or IL-6 (100 ng). Values expressed as a percentage of initial infection at 6 hpi. Values, mean ± SEM. *n* = 3 experiments. HFFs were infected with parasites and stained as described in the legend to Fig. 2. (B) Two-way ANOVA, Dunnett’s multiple-comparison test compared to WT_III_. ****, *P* ≤ 0.0001. ns = not significant. (C) One-way ANOVA, Dunnett’s multiple-comparison test compared to WT_III_. *, *P* ≤ 0.05; ****, *P* ≤ 0.0001. ns = not significant. Download FIG S4, TIF file, 0.7 MB. Copyright © 2021 Kochanowsky et al. 2021 Kochanowsky et al. https://creativecommons.org/licenses/by/4.0/ This content is distributed under the terms of the Creative Commons Attribution 4.0 International license.

**FIG 5:**
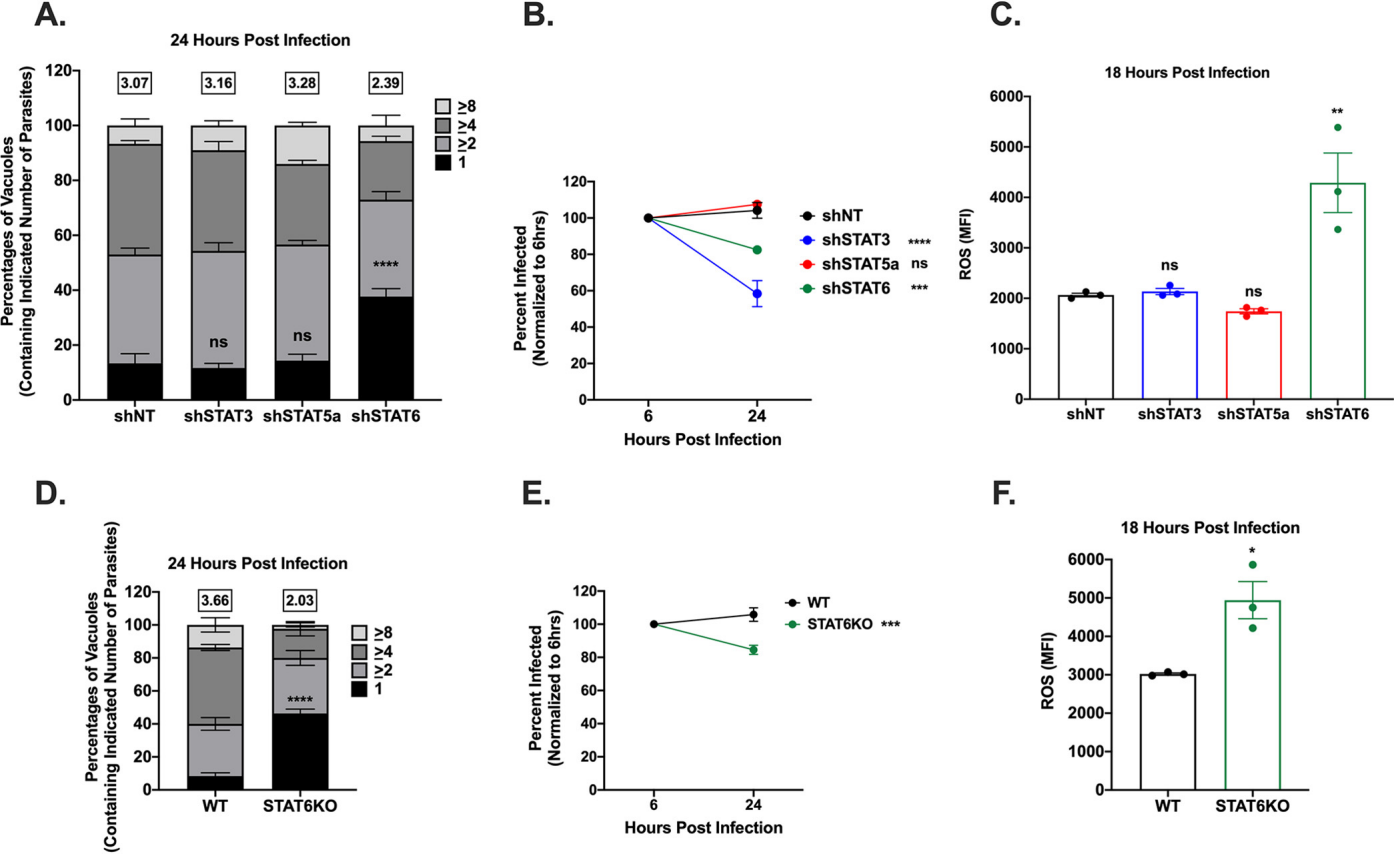
Suppression of STAT6 restricts parasite growth and survival. (A) Replication of WT_III_ in knockdown HFFs at 24 hpi. Mean percentages of parasite vacuoles containing indicated number of parasites. Numbers above stacked bar graph represent the overall mean number of parasites per vacuole. Bars, mean ± SEM. *n* ≥ 100 vacuoles/well, 3 wells/experiment, 3 experiments. (B) Intracellular survival of WT_III_ in knockdown HFFs 24 hpi expressed as a percentage of initial infection at 6 hpi. HFFs were infected with parasites and stained as in Fig. 2. Values, mean ± SEM. *n* = 3 experiments. (C) Quantification of ROS in knockdown HFFs. Each dot = mean ROS value for 1 experiment. *n* = 100 cells/experiment, 3 experiments. Bars, mean ± SEM. (D) Replication of WT_III_ in WT or STAT6KO mETFs at 24 hpi. Mean percentages of parasite vacuoles containing indicated number of parasites. Numbers above stacked bar graph represent the overall mean number of parasites per vacuole. Bars, mean ± SEM. *n* ≥ 100 vacuoles/well, 3 wells/experiment, 3 experiments. (E) Intracellular survival of WT_III_ in WT or STAT6KO mETFs at 24 hpi expressed as a percentage of initial infection at 6 hpi. HFFs were infected with parasites and stained as in Fig. 2. Values, mean ± SEM. *n* = 3 experiments. (F) Quantification of ROS in WT or STAT6KO mETFs as in Fig. 3. Each dot = mean ROS value for 1 experiment. *n* = 100 cells/experiment, 3 experiments. Bars, mean ± SEM. (A and D) Two-way ANOVA, Dunnett’s multiple-comparison test compared to shNT or WT. ****, *P* ≤ 0.0001. ns = not significant. (B, C, E, and F) One-way ANOVA, Dunnett’s multiple-comparison test compared to shNT or WT. *, *P* ≤ 0.05; **, *P* ≤ 0.005; ***, *P* ≤ 0.0005; ****, *P* ≤ 0.0001. ns = not significant.

To determine which STAT was involved in ROS production, we generated STAT3, STAT5a, and STAT6 knockdown HFF cell lines. We transduced HFFs with lentiviral vectors encoding shRNAs targeting STAT3, STAT5a, or STAT6 and then isolated single cells to establish clonal cell lines. We used Western blot analysis to determine knockdown efficiency and verify that only the targeted STAT was affected. We achieved knockdown efficiency of up to 60 to 80% of the targeted STAT (Fig. S5). We then tested growth and survival of WT_III_ parasites in these cell lines. Only the STAT6 knockdown cell line showed a growth and survival defect comparable to previous results with IIIΔ*rop16* (Fig. 5A and B). Interestingly, the STAT3 knockdown cells displayed a strong defect in parasite survival but not parasite growth (Fig. 5A and B). This finding is consistent with a recent report demonstrating that early ROP16-independent activation of STAT3 during the invasion process of host cells prevents parasite targeting by autophagy (45). As this finding is ROP16 independent and does not explain the growth defect found in our IIIΔ*rop16* strains, we reasoned that lack of STAT6 activation was more likely to explain the ROS-mediated killing effect in HFFs.

10.1128/mBio.03305-20.5 FIG S5 Generation of STAT3, 5a, and 6 knockdown cell lines. Top, representative Western blot from HFF cell lines with STAT-specific knockdowns (or nontargeting control [shNT]). Bottom, densitometry of total STAT3/5a/6 normalized to actin loading control. Bars, mean ± SEM. Each dot = 1 experiment. Download FIG S5, TIF file, 0.6 MB. Copyright © 2021 Kochanowsky et al. 2021 Kochanowsky et al. https://creativecommons.org/licenses/by/4.0/ This content is distributed under the terms of the Creative Commons Attribution 4.0 International license.

To test whether STAT6 suppression results in ROS accumulation during infection, we infected our knockdown cell lines with WT_III_ parasites and quantified host cell ROS levels. We observed the accumulation of ROS specifically in the STAT6 knockdown cells but not in the STAT3 or STAT5a knockdown cell lines (Fig. 5C). To determine if STAT6-mediated ROS accumulation also played a role in mouse cells, we generated mETFs from STAT6KO mice and infected them with WT_III_ parasites. Deletion of STAT6 from mETFs resulted in a defect in parasite growth and survival and an accumulation of ROS in infected host cells (Fig. 5D to F). Together, these data indicate that activation of STAT6 is required for suppression of host cell ROS production, which enables efficient type III parasite growth and survival in both human and mouse fibroblasts.

### Pharmacological suppression of NOX2- and mitochondrially derived ROS is sufficient to restore parasite growth and survival.

**FIG 6:**
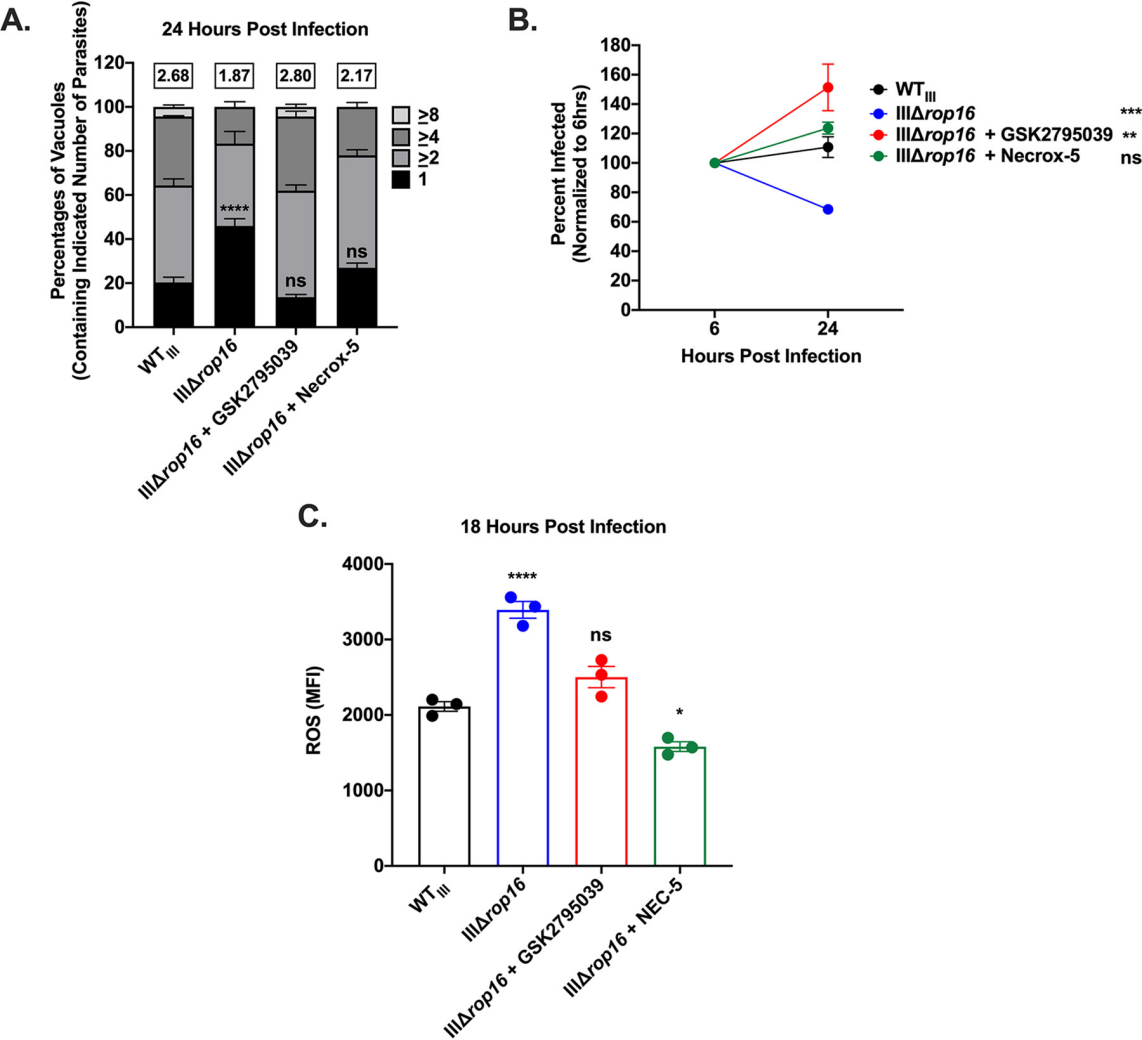
NOX2- and mitochondrially derived ROS restrict parasite growth and survival. (A) Replication of indicated strains in HFFs ± treatment with a NOX2 inhibitor, GSK2795039, or an mROS scavenger, Necrox-5, for 24 h during infection. Mean percentages of parasite vacuoles containing indicated number of parasites. Numbers above stacked bar graph represent the overall mean number of parasites per vacuole. Bars, mean ± SEM. *n* ≥ 100 vacuoles/well, 3 wells/experiment, 3 experiments. (B) Intracellular survival of indicated strains ± treatment with GSK2795039 or Necrox-5 for 24 h during infection in HFFs expressed as a percentage of initial infection at 6 hpi. HFFs were infected with parasites (MOI 1) and stained as described in the legend to Fig. 2. Values, mean ± SEM. *n* = 3 experiments. (C) Quantification of ROS as in Fig. 3 in HFFs ± treatment with GSK2795039 or Necrox-5 for 18 h during infection. Each dot = mean ROS value for 1 experiment in the indicated group. *n* = 100 cells/experiment, 3 experiments. Bars, mean ± SEM. (A) Two-way ANOVA, Dunnett’s multiple-comparison test compared to WT_III_. ****, *P* ≤ 0.0001. ns = not significant. (B and C) One-way ANOVA, Dunnett’s multiple-comparison test compared to WT_III_. *, *P* ≤ 0.05; **, *P* ≤ 0.005; ***, *P* ≤ 0.0005; and ****, *P* ≤ 0.0001. ns = not significant.

A recent study demonstrated that type III strains are cleared from naive murine bone marrow-derived macrophages (BMDMs) via NADPH oxidase 2 (NOX2) (27). Additionally, it is known that mitochondrially derived ROS (mROS) are capable of aiding in the clearance of bacteria (46, 47). To test whether NOX2-derived ROS or mROS played a role in parasite restriction, we infected confluent monolayers of HFFs pretreated with either GSK2795039, a NOX2-specific inhibitor (48), or Necrox-5, an mROS scavenger (47), with IIIΔ*rop16* parasites and assayed growth, survival, and ROS generation. Treatment of HFFs with either GSK2795039 or Necrox-5 was sufficient to restore growth and survival of IIIΔ*rop16* parasites to WT_III_ levels (Fig. 6A and B). Additionally, treatment of HFFs with either GSK2795039 or Necrox-5 was sufficient to lower host cell ROS accumulation during IIIΔ*rop16* infection to WT_III_ levels (Fig. 6C). Taken together, these results indicate that both NOX2-derived ROS and mROS play a role in restriction of IIIΔ*rop16* parasites.

## DISCUSSION

The data presented here show that one well-studied polymorphic effector protein, ROP16, is responsible for suppressing IFN-γ-independent production of ROS, allowing for efficient parasite growth and survival of an avirulent type III strain of T. gondii in HFFs. We have shown that efficient survival of the type III strain during its lytic life cycle in HFFs requires a ROP16 that has a functional kinase domain and can activate STATs. We also observed that ROP16-mediated suppression of ROS occurred in other host backgrounds (mouse) and other cell types (macrophages). Using a combination of cytokine stimulation and HFF STAT knockdown or mETF STAT6KO cell lines, we determined that ROP16-dependent activation of STAT6 is required to abrogate ROS-mediated restriction of type III parasite growth and survival. Finally, using pharmacological inhibitors we showed that both NOX2 and mitochondria are sources of ROS that restrict type III parasites in the absence of ROP16_III_. These findings highlight a role for ROS as an IFN-γ-independent mechanism of parasite control that is suppressed by ROP16_III_ in human cells.

Though we have identified a previously unrecognized role for ROP16 in repression of host cell ROS production, we have not identified what host-parasite interactions trigger the production of ROS that is ultimately suppressed by ROP16_III_. Interestingly, we observed that uninfected cells in these cultures showed a trend toward an increased accumulation of ROS compared to uninfected cells in cultures with WT_III_ parasites. Given that we have previously shown that T. gondii injects its rhoptry contents into cells it does not invade (26), these data may indicate that injection of ROP proteins without ROP16 triggers the host cell ROS response. Another possibility is that the actively infected cells release a signal that has a paracrine effect that produces ROS in neighboring uninfected cells.

While we have not determined what host-parasite interactions trigger the production of ROS, we have identified both NOX2 and mitochondria as important sources of ROS generation. NADPH oxidases are known to drive the respiratory burst that kills phagocytosed pathogens (49). Though we have not performed electron microscopy studies to show the exact mechanism by which the ROS kill parasites, we anticipate that such studies would be consistent with the transmission electron microscopy studies done in a recent paper that implicated NADPH oxidase in the control of intracellular type III parasites in naive murine macrophages (27). The NADPH oxidase-dependent effect in this prior study identified a ROP16-independent effect, highlighting that ROS killing mechanisms may differ between immune cells (macrophages) and non-immune cells (fibroblasts) and between hosts (murine versus human).

Our observation that mROS also played a role in parasite restriction is in keeping with several studies that have shown that mROS plays an important role in macrophage killing of phagosomally localized bacteria (46, 47) and extends this work to suggest that mROS may play in important role in pathogen defense in nonimmune cells as well. This finding is especially interesting given that type I and III strains, but not type II strains, show high levels of host mitochondrion association with the PV, the host membrane-derived structure, which surrounds intracellular parasites and avoids fusion with the phagolysosome (50–59). Host mitochondria associating with the PV might be able to deliver mitochondrion-derived vesicles harboring ROS (47) to the underlying parasites. Such a mechanism might also partially explain why strains that have host mitochondrial association also retain a ROP16 that can suppress ROS production. Additionally, at least one study has shown that STAT6 colocalizes with mitochondria (60) and thus may have other roles outside of acting as a transcription factor. Future studies will focus on the link between type I and III strains’ host cell mitochondrion association and STAT6-dependent suppression of host ROS generation.

While invasion of its host cell and establishment of the PV during T. gondii infection are processes that are largely conserved between strains, a growing body of research has shown that the repertoire of secreted effector proteins can have distinct functions between strains such as the activation of STATs by type I and III alleles of ROP16 and NF-κB activation by GRA15 in type II strains (20, 61–64). Notably, type II strains lack a ROP16 that can activate STATs, though it is predicted to have a functional kinase domain (20–24). This difference raises the question of how type II strains avoid ROS-mediated parasite restriction. A few possible explanations include that type II strains might not trigger an ROS response, or they might have a compensatory ROP16-independent mechanism that suppresses ROS production or an increased capacity for tolerating the presence of ROS. Interestingly, type II strains that lack ROP16 show no *in vivo* defect and may even cause an increase in CNS parasite burden (65). Together, these data highlight that host-specific pressures likely influenced the evolution of T. gondii strain-specific traits. Type III strains may have evolved to survive in hosts with particularly strong ROS responses. Future work should be directed toward determining what downstream STAT6-regulated genes are involved in blocking ROS production and identifying how differences in ROS production in T. gondii’s broad host range affect the survival of different T. gondii strains.

## MATERIALS AND METHODS

### Parasite maintenance.

As previously described, all parasite strains used in this study were maintained through serial passage in human foreskin fibroblasts (gift of John Boothroyd, Stanford University, Stanford, CA) using Dulbecco modified Eagle medium (DMEM), supplemented with 10% fetal bovine serum, 2 mM glutagro, and 100 IU/ml penicillin and 100 μg/ml streptomycin (cDMEM). In addition to the strains described below, the previously described type III (CEP), IIIΔ*rop16*, and ROP16_III_ strains expressing mCherry or tdTomato were also used (25).

### IC-21 and THP1 cell lines.

IC-21s (gift of Janko Nikolich-Zugich, University of Arizona) were maintained in RPMI 1640 + l-glutamine supplemented with 10% fetal bovine serum, and 100 IU/ml penicillin and 100 μg/ml streptomycin prior to downstream experiments. THP1s (gift of Felicia Goodrum, University of Arizona) were maintained in RPMI 1640 + l-glutamine supplemented with 10% fetal bovine serum, 1 mM sodium pyruvate, and 100 IU/ml penicillin and 100 μg/ml streptomycin prior to downstream experiments. THP1 monocyte-macrophage differentiation was induced as previously described prior to downstream experiments (39). Briefly, THP1s were added to glass coverslips on 24-well plates and treated with 100 nM 12-O-tetra-decanoylphorbol-13-acetate (TPA) for 24 h and then replaced with standard growth medium for 48 h, after which the differentiated macrophages were used in downstream experiments.

### Generation of plasmids containing ROP16 constructs.

All the primers used to make and validate the IIIΔ*rop16* strain are listed in Table S1 in the supplemental material. The ROP16 construct-expressing plasmids were constructed using a previously described FLAG-tagged ROP16-encoding vector and the In-Fusion mutagenesis kit and protocol (Takara, no. 638920) (25).

10.1128/mBio.03305-20.6 TABLE S1 List of primers used throughout the paper. Download Table S1, DOCX file, 0.02 MB. Copyright © 2021 Kochanowsky et al. 2021 Kochanowsky et al. https://creativecommons.org/licenses/by/4.0/ This content is distributed under the terms of the Creative Commons Attribution 4.0 International license.

### Generation of ROP16 mutant strains.

Generation of IIIΔ*rop16* and ROP16_III_ parasites was previously described (25). For the newly generated strains, IIIΔ*rop16* parasites were transfected with 50 μg of linearized plasmid DNA harboring FLAG-tagged ROP16 constructs and a bleomycin resistance marker. Selection for bleomycin resistance was performed as previously described (25, 66). Post-transfection freshly egressed parasites were resuspended in DMEM supplemented with 50 μg/ml of Zeocin (InvivoGen, 11006-33-0) for 4 h and then added to HFF monolayers supplemented with 5 μg/ml Zeocin to select for integrants. This process was repeated 3 times prior to plating by limiting dilution to isolate single clones. Single clones were subsequently screened by PCR for ROP16 integration (Fig. S1A; PCR primers listed in Table S1).

### Immunofluorescence microscopy.

For immunofluorescence assays, cells were fixed with 4% paraformaldehyde for 15 min. The cells were then permeabilized and blocked for 60 min with 0.1 to 0.2% (vol/vol) Triton X-100 in phosphate-buffered saline (PBS) (pH 7.4), typically with 3% (wt/vol) goat serum. For T. gondii intracellular growth and survival assays, cells were incubated with mouse polyclonal anti-SAG1 (DG52) (67) at 1:10,000 overnight, followed by incubation with Alexa Fluor 647 secondary antibodies (Molecular Probes) for 1 h. For GBP5 localization assays, cells were incubated with anti-SAG1 (DG52) at 1:10,000 and anti-GBP5 (Cell Signaling Technologies, no. 67798) at 1:500 overnight followed by incubation with Alexa Fluor 647 and Alexa Fluor 488 secondary antibodies (Molecular Probes) for 1 h. For ROP16 localization assays, cells were incubated with anti-SAG1 (DG52) at 1:10,000 and anti-DYKDDDDK (Cell Signaling Technologies, no. 14793) at 1:500 overnight followed by incubation with Alexa Fluor 647 and Alexa Fluor 488 secondary antibodies (Molecular Probes) for 1 h. Coverslips were mounted on slides using Fluoromount G (ThermoFisher, 00-4958-02).

### Western blotting.

Parasites (multiplicity of infection [MOI] 5) were added to HFFs, and protein was extracted at 24 hpi as previously described (20). Western blot analyses were performed using antibodies specific for total STAT3, STAT5a, and STAT6 and for the phosphorylated forms of STAT3 (phospho-Tyr705), STAT5a (phospho-Tyr694), and STAT6 (phospho-Tyr641) (Cell Signaling Technologies; catalog numbers 9139, 94205, 5397, 9145, 9314, and 56554). Anti-beta-actin was used as a loading control (Cell Signaling Technologies, no. 3700). For cytokine assays, HFFs were treated with 100 ng of recombinant human IL-4 or IL-6 (Sigma-Aldrich numbers I4269 and I2786) for 6 h before protein extraction and Western blot analysis. All blots were imaged on an Odyssey CLx (Li-COR). Densitometry was performed using ImageJ/FIJI as previously described (68).

### Plaque assay.

Confluent monolayers of HFF cells were infected with 200 tachyzoites of the indicated strains in cDMEM, and cultures were left undisturbed for 10 days to allow for development of plaques, after which the media in the cultures was aspirated, and the cultures were fixed in ice-cold 100% methanol for 10 min. Fixed monolayers were stained with crystal violet for 15 min at room temperature. Stained monolayers were then analyzed by light microscopy at ×4 magnification for the number of plaques in the HFF monolayers.

### T. gondii intracellular growth assays.

For the growth assay, parasite growth rate was determined using previously described methods which employ a direct parasite-per-vacuole scoring approach (69). Briefly, freshly syringe-lysed parasites (MOI 1) were added to confluent HFF monolayers grown on glass coverslips and spun down at 300 rpm for 1 min. After 1 h, the cultures were then washed to remove noninvaded parasites. At 24 hpi, infected monolayers were fixed, permeabilized, and stained with anti-SAG1 antibodies (as above) and then analyzed by fluorescence microscopy to identify the number of parasites per vacuole. One hundred vacuoles per coverslip were analyzed. For ROS rescue assays, HFFs were treated with either 5 mM N-acetyl-l-cysteine (Abcam, ab139473), 25 μM GSK2795039 (MedChemExpress, 1415925-18-6), or 10 μM Necrox-5 (Cayman Chemical, no. 17278) at the time of infection for either 6 or 24 hpi. Cells were then fixed, stained, and quantified as above. For cytokine rescue assays, HFFs were treated with 100 ng of recombinant human IL-4 or IL-6 (Sigma-Aldrich numbers I4269 and #I2786) at the time of infection to more closely mimic ROP16 injection. Cells were then fixed, stained, and quantified as above.

### T. gondii intracellular survival assays.

As previously described, freshly syringe-lysed parasites (MOI 1) were added to confluent HFF monolayers grown on glass coverslips (32). Cells were infected for 6 h followed by 3 PBS washes to remove extracellular parasites. Cells were fixed every 6 hpi up to 24 hpi using 4% formaldehyde. Prior to permeabilization, cells were stained with wheat germ agglutinin (ThermoFisher W11261) conjugated to Alexa Fluor 488 according to manufacturer’s recommendations. Cells were then permeabilized and stained with anti-SAG1 antibodies and Alexa Fluor 647-conjugated secondary antibody as described above. Images were acquired at 40× on a Leica DMI6000 with a motorized stage, using Leica Application Suite X (LAS X). Data from at least 50 fields of view per experiment were used to calculate the percent decrease in infected cells at various time points versus 6 hpi. For ROS rescue assays, HFFs were treated with either 5 mM N-acetyl-l-cysteine (Abcam, ab139473), 25 μM GSK2795039 (MedChemExpress, 1415925-18-6), or 10 μM Necrox-5 (Cayman Chemical, no. 17278) at the time of infection for either 6 or 24 hpi. Cells were then fixed, stained, and quantified as above. For cytokine rescue assays, HFFs were treated with 100 ng of recombinant human IL-4 or IL-6 (Sigma-Aldrich I4269 and I2786) at the time of infection to more closely mimic ROP16 injection. Cells were then fixed, stained, and quantified as above.

### Intracellular ROS/RNS measurement assays.

Freshly syringe-lysed parasites (MOI 1) were added to confluent HFF monolayers grown on glass coverslips and allowed to infect for 18 h. Cells were then stained using a commercially available cellular ROS/RNS assay kit (Abcam, ab139473) following the manufacturer’s protocol. Cells were then imaged at 40× on a Leica DMI6000 with a motorized stage, using Leica Application Suite X (LAS X). One hundred cells per experiment over 50 fields of view were quantified using ImageJ/FIJI. Briefly, individual cells were selected by using the selection tools and the mean fluorescent intensity per cell was obtained by the measure tool as previously described (70, 71).

### GBP5 localization assays.

HFF monolayers grown on glass coverslips were treated with or without 100 U of IFN-γ (R&D Systems, 285-MI-100) for 24 h prior to infection. Cells were then infected for 9 h, after which they were fixed using 4% formaldehyde. Cells were then permeabilized and stained as described above. Images were acquired at 40× on a Leica DMI6000 with a motorized stage, using Leica Application Suite X (LAS X). Data from at least 50 fields of view per experiment were used to calculate the percent GBP5 localization around the PV.

### Generation of STAT3/5a/6 knockdown cell lines.

Vectors encoding shRNA targeting STAT3, STAT5a, and STAT6 and a nontargeting control were acquired from Sigma-Aldrich (SHCLNG-NM_003150-TRCN0000329887, SHCLNG-NM_003152-TRCN0000232134, SHCLNG-NM_003153-TRCN0000274209, and SHC002). HEK-293T cells (gifts of Deepta Bhattacharya, University of Arizona) were transfected with 1 μg of the respective shRNA-encoding plasmid, 750 ng of psPAX2 packaging plasmid, and 250 ng pMD2.G envelope plasmid (gifts of Felicia Goodrum, University of Arizona) using FuGENE transfection reagent and protocol (Promega, no. TM328). Resultant lentiviral particles were collected and concentrated at 24 and 48 hpi using Lenti-X concentrator reagent and protocol (Clontech, no. 631231). HFFs were transduced with 40 μl of concentrated lentiviral particles, and integrants were selected for with 3 μg of puromycin (InvivoGen, no.ant-pr-1) for 48 h. Cells were subjected to limiting dilution and then expanded prior to Western blot analysis.

### Generation of murine fibroblasts.

B6.129S2(C)-Stat6^tm1Gru^/J (STAT6KO) mice were purchased from Jackson Laboratories (stock no. 005977) and bred in the University of Arizona BIO5 Animal Facility in accordance with protocols approved by the University of Arizona Institutional Animal Care and Use Committee. Murine ear tip fibroblasts (mETFs) were generated according to previously described protocols (72). Briefly, 2-mm ear punch biopsy specimens were collected from the pinna of adult mice, submerged in 70% ethanol, and then transferred to mETF culture medium on ice for dissection. Tissue layers were dissected apart, cut into 1-mm by 1-mm pieces, and then transferred to a 6-well gelatin-coated plate containing mETF medium. Tissue pieces were evenly distributed and pressed to the bottom of the wells. Fibroblast outgrowth took approximately 2 weeks to reach confluence, after which mETFs were passaged to 100-mm plates for expansion. mETFs were cultured in DMEM supplemented with 20% fetal bovine serum, 100 IU/ml penicillin, 100 μg/ml streptomycin, 1× HEPES, and 1× minimal essential medium with non-essential amino acids. After first passage, culture medium was reduced to 10% fetal bovine serum for line maintenance. To generate WT mETFs, the same procedure was used on ear punch biopsy specimens from C57BL/6 (WT) mice purchased from Jackson Laboratories (stock no. 000664).

### Statistical analysis.

Graphs were generated and statistical analyses were performed using Prism 8.4.2 software. All experiments were performed at least three independent times, and statistical analyses were conducted on the composite data unless reported otherwise. Unless otherwise specified, the data were analyzed using a one-way analysis of variance (ANOVA) with Dunnett’s multiple-comparison test to the control (WT_III_). For T. gondii intracellular growth assays, a two-way ANOVA with Dunnett’s multiple-comparison test to the control (WT_III_) was used.

### Data availability.

All relevant data are within the paper and its supplemental material. All strains, primers, and plasmids are available upon request.
