# Supplementary figures and images for: ROP16-Mediated Activation of STAT6 Suppresses Host Cell Reactive Oxygen Species Production, Facilitating Type III Toxoplasma gondii Growth and Survival
Source: mBio. 2021 Mar 2;12(2):e03305-20. doi: 10.1128/mBio.03305-20 (PMC8092286; doi:10.1128/mBio.03305-20)

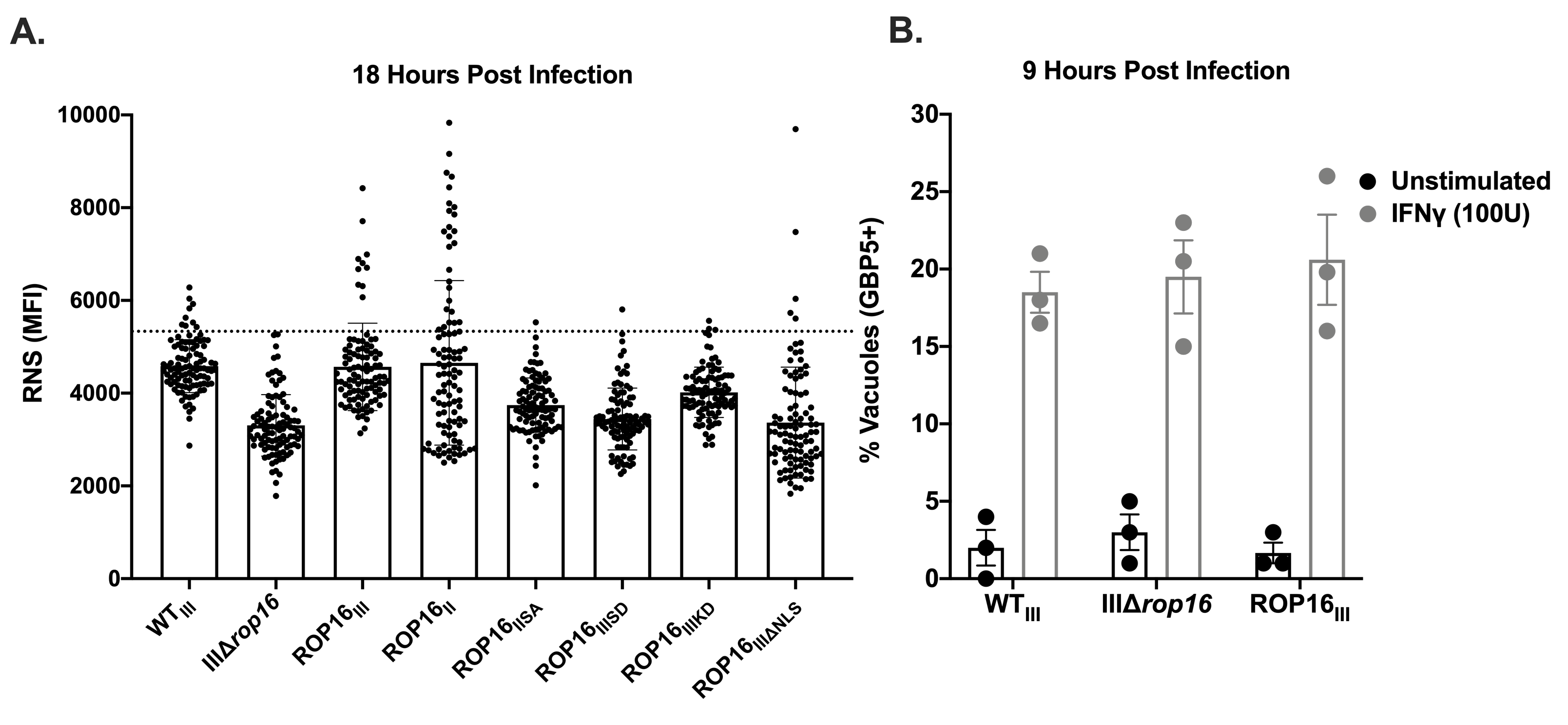

Supplement: FIG S2 [file mBio.03305-20-sf002.tif]

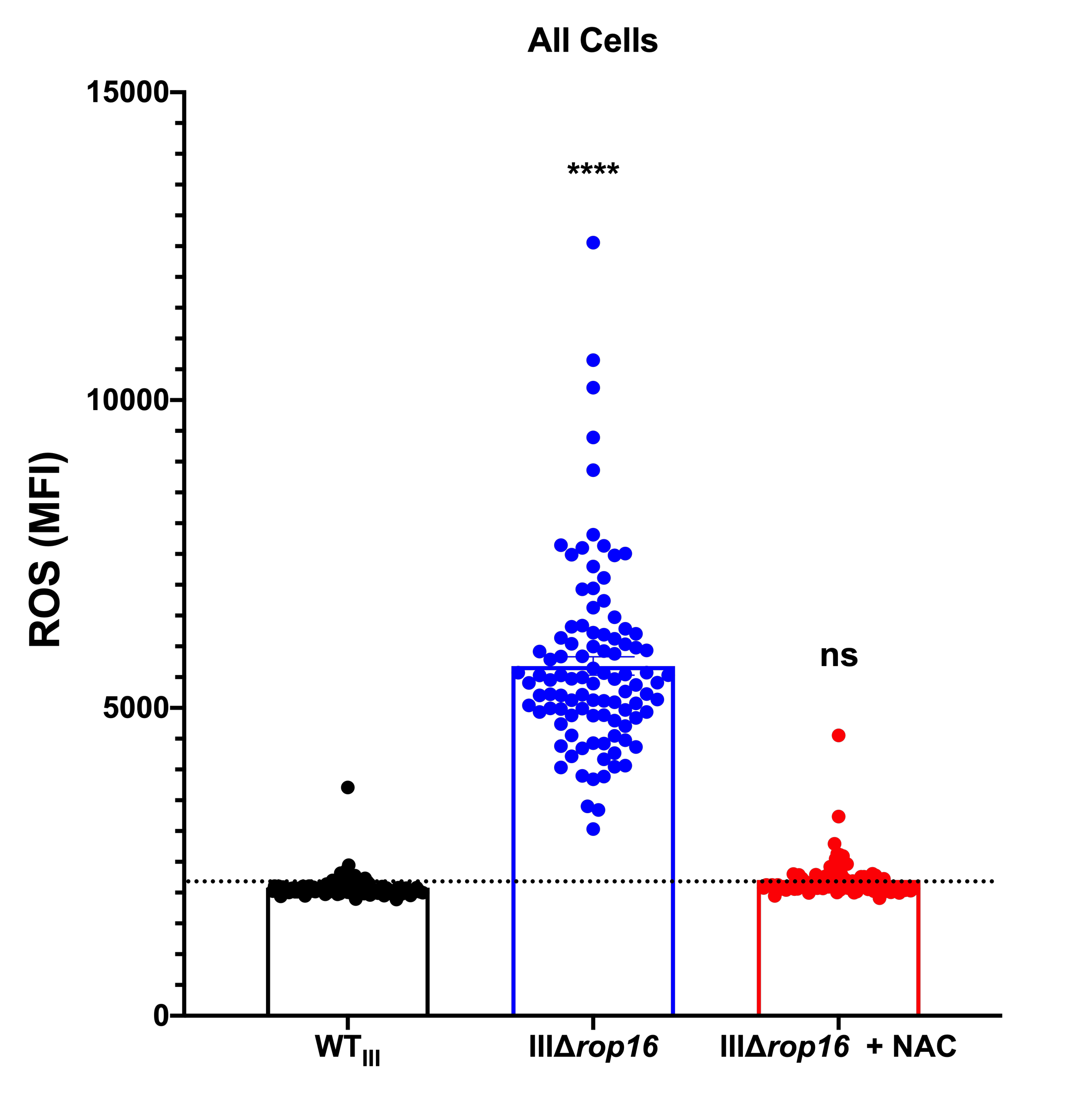

Supplement: FIG S3 [file mBio.03305-20-sf003.tif]

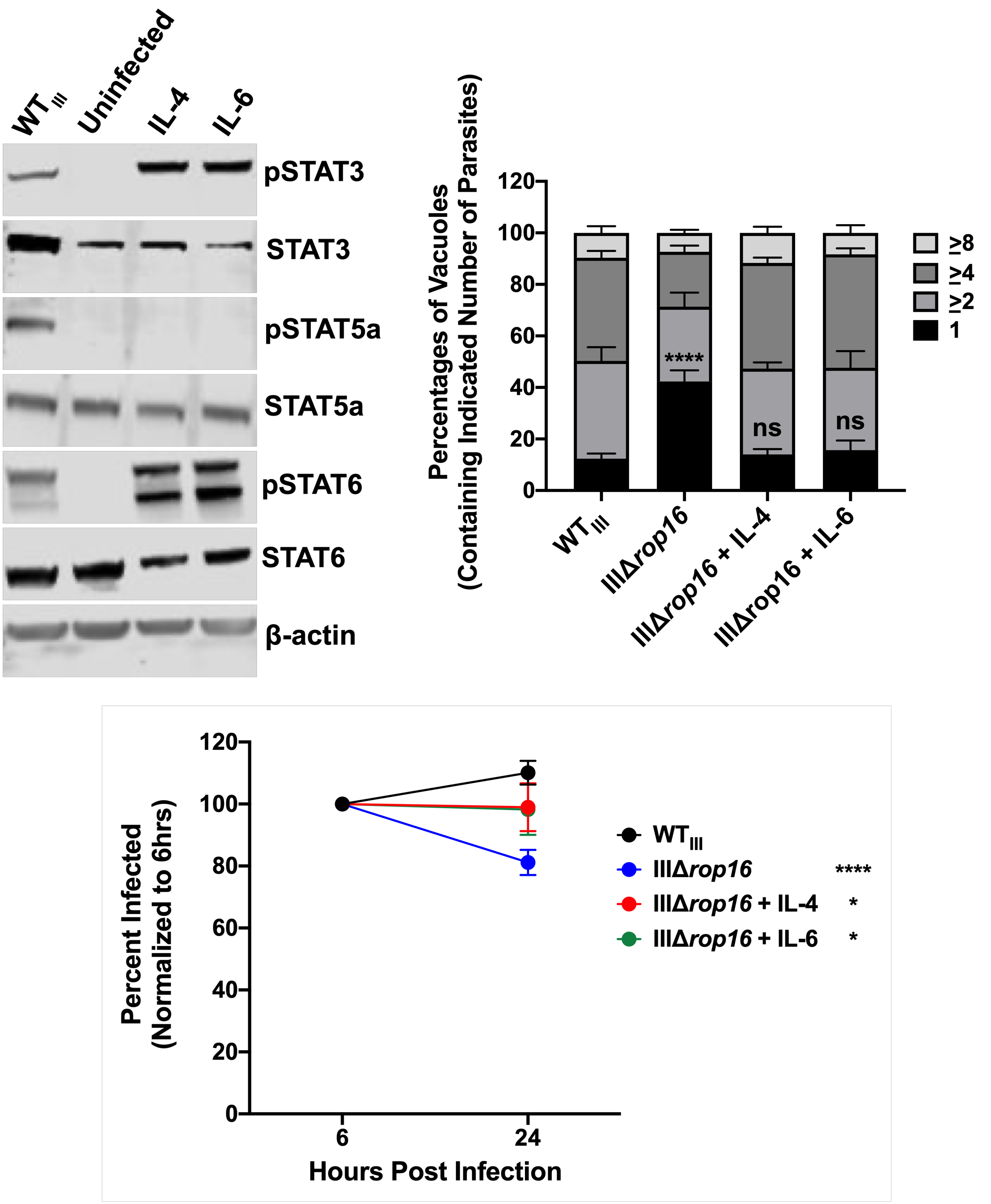

Supplement: FIG S4 [file mBio.03305-20-sf004.tif]

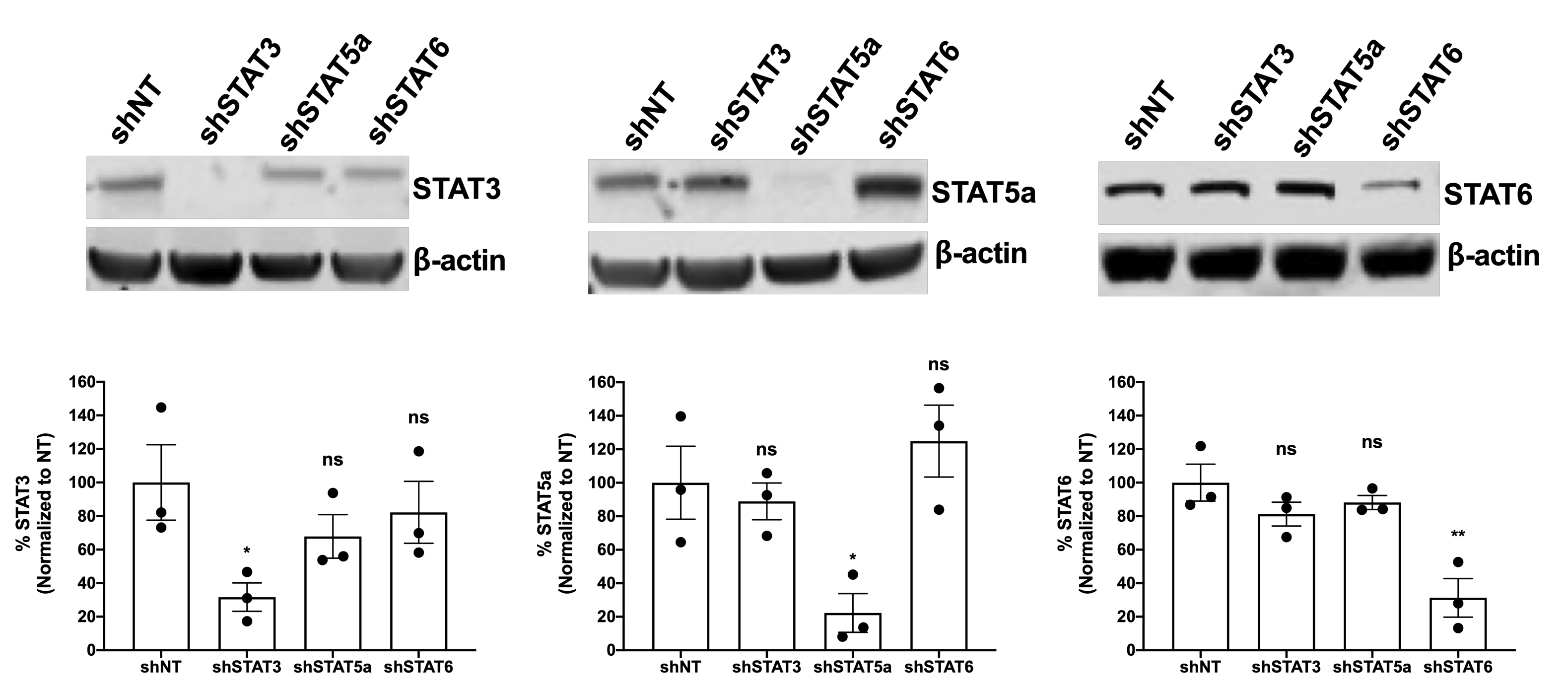

Supplement: FIG S5 [file mBio.03305-20-sf005.tif]
