# Supplementary material for: ROP16-Mediated Activation of STAT6 Suppresses Host Cell Reactive Oxygen Species Production, Facilitating Type III Toxoplasma gondii Growth and Survival
Source: mBio. 2021 Mar 2;12(2):e03305-20. doi: 10.1128/mBio.03305-20 (PMC8092286; doi:10.1128/mBio.03305-20)
Supplement: TABLE S1 [file mBio.03305-20-st001.docx]

**Table S1. List of Primers used throughout the paper.**

| **Primers** | **Sequences** |
| --- | --- |
| SagI Sequencing FWD Set 4 | CGCTGCACCACTTCATTATTT |
| SagI Sequencing REV Set 4 | TGTTCCCGCAGACGATTT |
| Rop16 Internal CDS FWD | GGAGTTGGATGTTCGGGATAAG |
| Rop16 Internal CDS REV | GCTCTTGAGGGTCTGGATTTAG |
| Rop16-FLAG Bleo In Fusion FWD | ACAAAGACGATGACGACAAGTAGGGTGTAAGGTTCCCACC |
| Rop16-FLAG Bleo In Fusion REV | CGTCATCGTCTTTGTAGTCCATCCGATGTGAAGAAAGTTCGG |
| Rop16NLS-FLAG Bleo In Fusion FWD | GTATGCGGATGAGAATGCAACCTCCAGGAGCGGTG |
| Rop16NLS-FLAG Bleo In Fusion REV | TTCTCATCCGCATACGGGCAGCAACCCTCTGTTCTG |
| Rop16KD-FLAG Bleo In Fusion FWD | TGCAGCGAATGTGCCGTACAGCCAGATCG |
| Rop16KD-FLAG Bleo In Fusion REV | GGCACATTCGCTGCATACAGCGTCCC |
| Rop16SD-FLAG Bleo In Fusion FWD | CTCCGCATCGAACAGTCTAGTCCAGTCGCAACC |
| Rop16SD-FLAG Bleo In Fusion REV | CTGTTCGATGCGGAGCCATCAATTAATGGC |
| Rop16SA-FLAG Bleo In Fusion FWD | CTCCGCATTGAACAGTCTAGTCCAGTCGCAACC |
| Rop16SA-FLAG Bleo In Fusion REV | CTGTTCAATGCGGAGCCATCAATTAATGGC |
